# A statistical survey of ultralow‐frequency wave power and polarization in the Hermean magnetosphere

**DOI:** 10.1002/2016JA023103

**Published:** 2016-09-28

**Authors:** Matthew K. James, Emma J. Bunce, Timothy K. Yeoman, Suzanne M. Imber, Haje Korth

**Affiliations:** ^1^ Department of Physics and AstronomyUniversity of LeicesterLeicesterUK; ^2^ Department of Atmospheric, Oceanic and Space SciencesUniversity of MichiganAnn ArborMichiganUSA; ^3^ The Johns Hopkins University Applied Physics LaboratoryLaurelMarylandUSA

**Keywords:** ULF, waves, Mercury, MESSENGER

## Abstract

We present a statistical survey of ultralow‐frequency wave activity within the Hermean magnetosphere using the entire MErcury Surface, Space ENvironment, GEochemistry, and Ranging magnetometer data set. This study is focused upon wave activity with frequencies <0.5 Hz, typically below local ion gyrofrequencies, in order to determine if field line resonances similar to those observed in the terrestrial magnetosphere may be present. Wave activity is mapped to the magnetic equatorial plane of the magnetosphere and to magnetic latitude and local times on Mercury using the KT14 magnetic field model. Wave power mapped to the planetary surface indicates the average location of the polar cap boundary. Compressional wave power is dominant throughout most of the magnetosphere, while azimuthal wave power close to the dayside magnetopause provides evidence that interactions between the magnetosheath and the magnetopause such as the Kelvin‐Helmholtz instability may be driving wave activity. Further evidence of this is found in the average wave polarization: left‐handed polarized waves dominate the dawnside magnetosphere, while right‐handed polarized waves dominate the duskside. A possible field line resonance event is also presented, where a time‐of‐flight calculation is used to provide an estimated local plasma mass density of ∼240 amu cm^−3^.

## Key Points

- Evidence that the Kelvin‐Helmholtz instability is driving Hermean ULF wave activity
- Observations suggest that Earth‐like field line resonance is possible at Mercury
- Wave power mapped to Mercury's surface reveals the likely average polar cap boundary location

## 1 Introduction

### 1.1 ULF Wave Modes at Mercury

One of the first observations of ULF wave activity in the Hermean magnetosphere was found using magnetometer data obtained by Mariner 10 [*Russell*, 1989] during its first flyby of Mercury in 1974. In this event, wave activity with right‐handed (RH) circular polarization and a period of around 3 s was observed near the dawnside magnetopause and, as the spacecraft traversed deeper into the magnetosphere, the wave transformed into a narrowband, linearly polarized wave with a period of 2 s. The transition to a linearly polarized wave suggested that this may have been a resonance—*Russell* [1989] suggested that this wave could have been a fourth harmonic of the fundamental field line resonance (FLR) frequency, *f*_FLR_, based on some assumptions of field line length and Alfvén velocity, *v*_*A*_. Later it was argued by *Southwood* [1997] that this wave could not have been a pure FLR like those observed in the terrestrial magnetosphere as there was a significant compressional component to the wave, whereas terrestrial FLRs are shear Alfvén waves which oscillate predominantly azimuthally. Instead, *Southwood* [1997] suggested that these may be similar to standing waves at Earth modified by the presence of hot plasma [e.g., *Southwood*, 1976].

In the terrestrial magnetosphere, ultralow‐frequency (ULF) waves are standing waves with frequencies much lower than the local ion gyrofrequencies present in the magnetosphere (approximately mHz); therefore, they can be successfully described using the MHD (magnetohydrodynamic) treatment of waves used by *Dungey* [1963] and understood in terms of field line resonance as described above. In the Hermean magnetosphere, observed wave frequencies are typically of the same order as local ion gyrofrequencies (approximately Hz) [e.g., *Russell*, 1989]. The consequence of this is that the wave modes that can exist in such an environment cannot be described using the MHD treatment of waves and are likely to be related to the local gyroscopic motion of the plasma particles. This is because the time scales involved in Hermean ULF waves are so similar to those of the motion of individual plasma particles.

More recent observations at Mercury have demonstrated that it is indeed common to find wave activity with frequencies close to, but not exactly equal to, the proton gyrofrequency, *f*_cH+_ [e.g., *Boardsen et al.*, 2009a, 2009b; *Echer*, 2010; *Anderson et al.*, 2011b; *Boardsen et al.*, 2012], where the local proton gyrofrequency is typically in the range of 1 < *f*_cH+_<2 Hz. *Boardsen et al.* [2012] found that these waves were often accompanied by harmonics and that the most common peaks in wave power occurred in three places: a dominant peak just below *f*_cH+_, a second peak close to 2*f*_cH+_, and just below *f*_cHe++_. Waves often exhibited a mixture of transverse and compressional wave powers, where transverse wave power was typically dominant at high latitudes and compressional wave power peaked near the equator, though approximately a quarter of the events studied by *Boardsen et al.* [2012] were transverse at all latitudes. The total wave power also had a maximum near the equator, suggesting that there may be an equatorial source for these waves. Most of the waves observed by *Boardsen et al.* [2012] had a near‐linear polarization, where the handedness was most often RH (right handed), as previously observed by *Boardsen et al.* [2009a, 2009b]. *Kim and Lee* [2003] predicted that a RH polarized compressional mode would undergo a mode conversion where local gyroresonance is met, such that the energy would be transferred to a LH (left‐handed) polarized mode such as an ion‐cyclotron wave (ICW). If the fluctuations studied by *Boardsen et al.* [2009a, 2009b, 2012] were ICWs, then they should exhibit LH circular polarization and they should be guided along the background field, though what is actually observed is a bias toward RH polarization—even in those events which are predominantly transverse and field guided. One possible explanation *Boardsen et al.* [2012] had for this was that they had observed field‐aligned resonances which are standing waves formed by ICWs, where the observed wave was actually a combination of two oppositely directed ICWs.

Further analysis of the ∼1 Hz waves undertaken by *Boardsen et al.* [2015] showed that the compressional waves observed by *Boardsen et al.* [2012] could be interpreted as ion‐Bernstein waves. Ion‐Bernstein waves with a small compressional component excited by a local instability propagate between the hemispheres around the magnetic equator, cycling between a highly compressional state at the equator and low compression at higher latitudes. The significant dominance in compressional waves in observations could be explained by the group velocity reducing near the equator, causing a pileup of compressional wave activity.

When considering the likely frequencies and origins of wave activity at Mercury, an important additional factor to consider is that the plasma is actually a multicomponent plasma, which introduces new resonance conditions. The Hermean plasma consists of H and He ions sourced from the solar wind, alongside various species of pickup ions (O, K, and Na) produced by sputtering from the planetary surface [*Lammer and Bauer*, 1997]. The oxygen and potassium contribution to the plasma is insignificant compared to that of the sodium pickup ions [*Cheng et al.*, 1987]. One new resonance that would be present in this plasma is the sodium ion‐cyclotron frequency, *f*_cNa+_, though *Boardsen and Slavin* [2007] had found no evidence for sodium ICWs using Mariner 10 data. The other new resonances that exist in such a multicomponent plasma are ion‐ion hybrid (IIH) resonances and Buchsbaum resonances [*Buchsbaum*, 1960] which lie in between each pair of ion gyrofrequencies. The IIH resonance occurs at the crossover frequency, *f*_CR_ [*Othmer et al.*, 1999; *Glassmeier et al.*, 2004], where the frequency depends upon the relative ion concentration ratio and is likely to lie between ∼6 mHz and 7 Hz in the Hermean magnetosphere, where magnetic field strength, |**B**|, varies between ∼10 and 400 nT. At *f*_CR_, where the RH, LH, and X (“extraordinary”) modes intersect, the plasma supports linearly polarized modes, one of which is strictly guided and analogous to the shear Alfvén mode of MHD [*Othmer et al.*, 1999]. The crossover frequency is likely to be a preferred frequency for field line resonance; the location of such a resonance depends on where *f*_CR_ coincides with the “critical coupling” (resonant mode) frequency. This is analogous to the resonant mode coupling in MHD, where a fast magnetosonic wave couples with the toroidal, shear Alfvén mode in Earth's magnetosphere [*Tamao*, 1965; *Southwood*, 1974; *Chen and Hasegawa*, 1974].

Wave modeling by *Kim et al.* [2008, 2013, 2015] showed that the fast compressional mode is efficiently coupled to the IIH resonance. The mode conversion generates strongly field‐guided waves near the magnetic equator, which then propagate toward higher latitudes. IIH waves are partially reflected at the Buschbaum resonance but can tunnel through the stop gap allowing the wave to exist on a global scale, potentially providing the linearly polarized transverse waves observed at high latitudes by *Boardsen et al.* [2012].

### 1.2 ULF Wave Sources at Mercury

At Earth, ULF waves are driven by sources of energy both internal and external to the magnetosphere. Global toroidal FLRs are frequently driven by Kelvin‐Helmholtz (K‐H) waves forming on the magnetopause which are transmitted into the magnetosphere as FMS (fast magnetosonic) waves. These FMS waves are partially reflected at a turning point in the magnetosphere, leaving evanescent waves to traverse deeper into the magnetosphere and couple with the Alfvén mode [*Tamao*, 1965; *Southwood*, 1974; *Chen and Hasegawa*, 1974]. Kelvin‐Helmholtz surface waves with periods ranging from 10 to 70 s have been observed at the magnetopause at Mercury [e.g., *Boardsen et al.*, 2010; *Sundberg et al.*, 2010, 2012a] using MESSENGER (MErcury Surface, Space ENvironment, GEochemistry, and Ranging) magnetometer data, though with a distinct preference for K‐H vortices forming on the duskside magnetosphere. The dawn‐dusk asymmetry was also present in global kinetic hybrid simulations [*Paral and Rankin*, 2013], where the lack of growth on the dawnside magnetosphere is likely due to the large magnetosheath ion gyroradii thickening the velocity shear layer, thus weakening the instability. As discussed above, the MHD treatment of ULF waves at Mercury is not necessarily appropriate as many waves observed are close to local ion gyrofrequencies, but K‐H waves may still provide a significant energy source for FLRs at frequencies *f*_FLR_ below the lowest ion gyrofrequency or in the form suggested by *Othmer et al.* [1999] where coupling occurs instead at the crossover frequency, *f*_CR_.

Other potential sources of energy for ULF wave activity in the Hermean magnetosphere through the interaction with the solar wind and the IMF include solar wind buffeting [*Baumjohann et al.*, 2006] and flux transfer events (FTEs) [e.g., *Slavin et al.*, 2012; *Imber et al.*, 2014]. Mercury's magnetosphere is relatively incompressible compared to other magnetospheres, such as the Earth's or Jupiter's [*Glassmeier et al.*, 2004]. The “stiffness” of the Hermean magnetosphere means that buffeting by the solar wind will induce oscillations, causing the entire magnetosphere to “ring.” FTEs have been shown to provide at least 30% of the flux transport required to drive Mercury's rapid substorm cycle [*Imber et al.*, 2014] and can occur quasiperiodically in large numbers as “FTE showers” with periodicities of 8–10 s [*Slavin et al.*, 2012]. Both these sources could provide opportunities for wave coupling at the frequencies *f*_FLR_ and *f*_CR_.

An additional complication when considering the possibility of resonant wave generation at Mercury is the boundary condition at the footprints of the field lines. In the terrestrial magnetosphere, the boundary conditions for the waves are provided by the highly conducting ionosphere; the ends of the field lines are anchored to the ionosphere in both hemispheres, each providing a reflection point for the standing waves. The boundary conditions for ULF waves are unclear at Mercury as there is no significantly conductive ionosphere to provide the reflection points along the field line. It has been suggested that the metallic core of Mercury may provide a similar boundary condition to the ionosphere at Earth due to its high conductivity [*Russell*, 1989; *Othmer et al.*, 1999], though it could be the case that the regolith on Mercury is too resistive to anchor the field line, but instead provides an open‐ended (antinode) boundary for wave reflection [*Blomberg*, 1997; *Glassmeier et al.*, 2004; *Blomberg et al.*, 2007].

In the terrestrial magnetosphere, wave‐particle interactions such as drift resonance and drift‐bounce resonance [*Southwood et al.*, 1969] with gradient‐curvature drifting clouds of energetic particles are often responsible for the occurrence of small‐scale, localized poloidal MHD waves [e.g., *Yeoman et al.*, 2008, 2010]. This instability is unlikely to develop at Mercury, as the magnetosphere may be too small to trap the energetic particles which would provide the instability [*Blomberg et al.*, 2007]. However, another instability is likely to be present at Mercury due to its small size; loss cones at Mercury are typically quite large, causing large holes in the velocity space distribution to form [*Schriver et al.*, 2011]. Holes in the velocity space distribution provide an instability capable of supplying energy for wave‐particle interactions, an instability which reduces in size with *L* shell [*Blomberg et al.*, 2007; *Boardsen et al.*, 2012, 2015]. Localized instabilities such as this, or the temperature anisotropies suggested by *Anderson et al.* [2011b], can generate ICWs and ion‐Bernstein waves (typically approximately Hz at Mercury) and may be responsible for the production of many of the waves previously observed at Mercury.

As discussed above, wave activity, such as ICW, ion‐Bernstein waves, and IIH waves, with frequencies ∼1 Hz appears in a number of case studies and has been studied extensively by, for example, *Boardsen et al.*, 2012 [2012, 2015]. The K‐H instability, FTE showers, and solar wind buffeting could provide energy for much lower frequency waves in the tens to hundreds of mHz range, below the lowest cyclotron frequencies present at Mercury. Such wave sources could then lead to resonant wave coupling at the frequencies *f*_FLR_ and *f*_CR_.

ULF waves have been related to various properties of the terrestrial magnetic environment and may be useful in providing similar information about Mercury. *Takahashi et al.* [2014] used field line resonance observations by Geotail to determine plasma mass densities in the outer magnetosphere using a time‐of‐flight approximation integral which relates the plasma mass density to the period of a standing Alfvén wave. This relationship between plasma mass density and wave period could be used at Mercury to provide mass density estimates if Alfvén waves are present in the Hermean magnetosphere. Monochromatic Pc5–Pc6 pulsations have been shown to exist on closed field lines, equatorward of the terrestrial polar cap boundary [*Ables et al.*, 1998; *Lanzerotti et al.*, 1999; *Mathie et al.*, 1999; *Scoffield et al.*, 2007; *Pilipenko et al.*, 2015], and similar standing wave activity could be useful in identifying the location of a polar cap boundary at Mercury. The damping of terrestrial ULF waves is largely due to ionospheric Joule dissipation, the rate of which is determined by the conductivity at the footprints of the wave [*Newton et al.*, 1978], so it may also be possible to use wave activity at Mercury to provide an estimate of conductivity.

Here we present the first major statistical survey of wave activity in the range *f* < 0.5 Hz, to investigate the possible wave modes and sources below the cyclotron frequency. We employ the entire collection of MESSENGER (MErcury Surface, Space ENvironment, GEochemistry, and Ranging) magnetometer (MAG) [*Anderson et al.*, 2007] data from 23 March 2011 to 30 April 2015, in order to quantify the observed wave activity and to evaluate the importance of various proposed wave modes and wave source mechanisms.

## 2 Data

### 2.1 Magnetometer Data

Due to MESSENGER's highly elliptical orbit, only around one fifth to one third of the orbit is within the magnetosphere [*Anderson et al.*, 2007]. As this study is focused on magnetospheric waves, any data relating to the solar wind or magnetosheath were separated from the magnetospheric data and discarded. In order to determine whether the data were collected from within the magnetosphere, we used the list of magnetopause crossings provided by *Winslow et al.* [2013], which extends from 23 March 2011 to 19 December 2011, for the first 9 months of magnetometer data. The remaining magnetopause crossings were determined using the same method as that used by *Winslow et al.* [2013], where magnetopause boundary crossings were typically characterized by a sudden rotation in the measured field or a change in the character of the fluctuations in the field.

The remaining magnetospheric data are rotated into a coordinate system based upon the local ambient magnetic field, where one component lies parallel to the direction of the magnetic field, *B*_∥_, an azimuthal component, *B*_*ϕ*_, positive eastward, and the poloidal component which completes the right‐handed set, *B*_*P*_, is in the direction of the local radius of curvature of the field line. In order to perform this rotation, we use the KT14 magnetic field model for Mercury [*Korth et al.*, 2015] which is discussed in more detail in section 2.3.

### 2.2 Wave Detection

In order to study wave activity, Fourier analysis was performed on each component of the magnetic field data from each pass of MESSENGER through Mercury's magnetosphere using a sliding window of length 120 s. Typically, the MAG data are sampled at 20 Hz which allows the detection of wave frequencies up to 10 Hz. *Boardsen et al.* [2012] used a 20 s window to study ∼1 Hz waves; our use of a 120 s window allows us to study waves with much lower frequencies. For the purposes of this study, we are focusing on the lower frequency waves (*f* < 0.5 Hz). This frequency range excludes proton cyclotron waves from our study, leaving wave activity which may be related to heavy ion instabilities [*Glassmeier*, 1997; *Ip*, 1987], Kelvin‐Helmholtz waves [*Boardsen et al.*, 2010; *Sundberg et al.*, 2010, 2012b], and fundamental eigenmodes [*Russell*, 1989].

**Figure 1:**
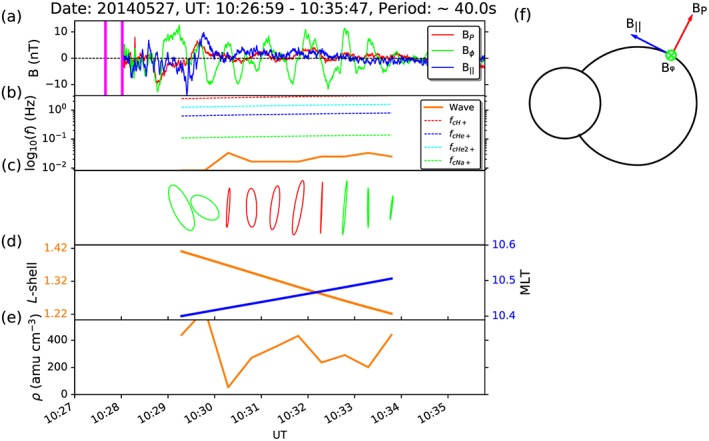
An example ULF wave detected using the MESSENGER MAG data. (a) The magnetometer data after rotation into the coordinate system described in section 2.1 and depicted in Figure 1f, where the poloidal (*P*), azimuthal (*ϕ*), and parallel (∥) components are in red, green, and blue, respectively. Pink vertical lines show the approximate range of time when MESSENGER transited through the magnetopause. (b) The detected frequency (in orange) on a logarithmic scale compared to the local ion‐cyclotron frequencies (red, blue, cyan, and green dashed lines). (c) The transverse polarization ellipses varying with time, where the vertical axis represents the azimuthal component and the horizontal axis represents both time and the poloidal component. The color of the ellipses represents their handedness, where green is left handed and red is right handed. (d) The *L* shell and magnetic local time (MLT) of MESSENGER's equatorial footprint as it moves through the magnetosphere. (e) The estimated plasma mass density, *ρ*, based on field line length and wave frequency.

Figure 1a shows an example of ULF wave activity detected by MAG shortly after MESSENGER entered the dayside magnetosphere between 10:27 and 10:36 UT on 27 May 2014. The data in this figure are presented in the coordinate system described above and depicted by Figure 1f, where the poloidal, azimuthal, and parallel components of the magnetic field are red, green, and blue, respectively. The frequency of this wave is indicated in Figure 1b in orange (∼25 mHz), and is lower than that of the local ion gyrofrequencies of H^+^, He^+^, He^2+^, and Na^+^ represented by green, blue, cyan, and red dashed lines, respectively.

**Figure 2:**
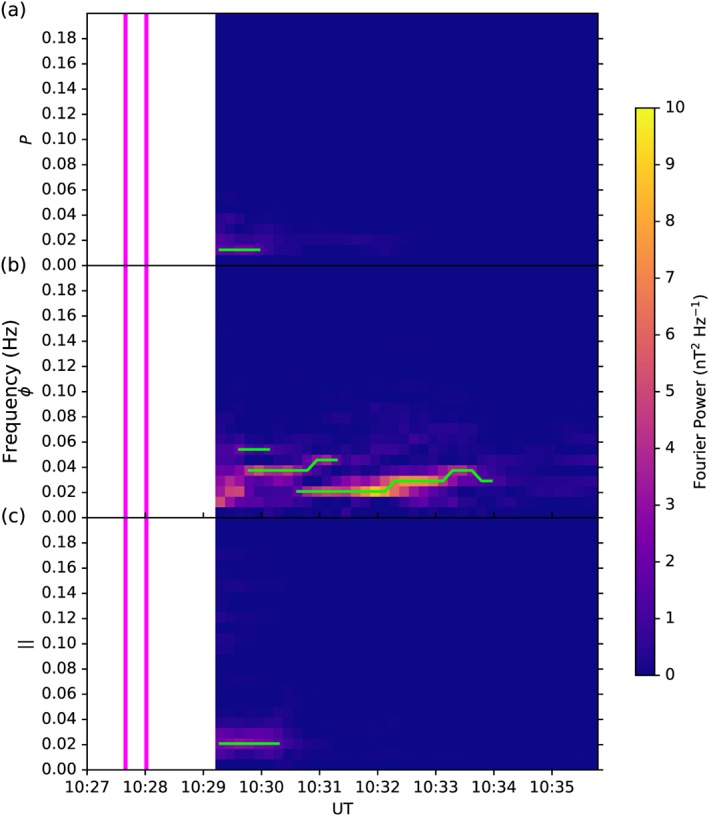
The spectrogram of the example waves in Figure 1, showing wave power for frequencies below 0.2 Hz as a function of time for (a) the poloidal, *P*, component, (b) the azimuthal, *ϕ*, component, and (c) the parallel, ∥, component. Yellow signifies higher wave power, and green lines show where the wave activity was detected by our routine. The two vertical pink lines show approximately when MESSENGER traversed through the magnetopause, into the magnetosphere.

In order to detect the wave activity, we evaluated the peaks and troughs within each power spectrum. The spectral peaks were compared to their neighboring troughs, where they were kept if their peak power was at least 1.4 times the power of both troughs. The value of 1.4 was determined by visually comparing a range of different multipliers, where lower values were able to detect smaller peaks in wave power, and larger values only detected the largest, most significant peaks in the power spectra. Figure 2 shows the corresponding Fourier power spectra for each component of the example wave presented in Figure 1a, where Figure 2a shows the poloidal (*P*) wave power, Figure 2b shows the azimuthal (*ϕ*) wave power, and Figure 2c shows the parallel (∥) wave power shortly after MESSENGER enters the magnetosphere through the magnetopause (shown as pink vertical lines). High wave powers appear yellow/orange in these spectrograms, and the waves detected are identified by green traces. It is clear from both the magnetometer traces and the spectrograms that this wave exhibits a significant azimuthal component (green), particularly from 10:30 to 10:34 UT, where the other components have much lower wave powers.

The complex output of the fast Fourier transform (FFT) is used to derive various wave characteristics, such as the Fourier phase. Using the method described by *Born and Wolf* [1980], the wave amplitudes and Fourier phases for the two transverse magnetic field components (*P* and *ϕ*) can be used to determine the eccentricity, *e*, of the transverse polarization ellipse at any given frequency. For purely circularly polarized waves, *e* = 0, and for linearly polarized waves, *e* = 1. Figure 1c shows the polarization ellipses calculated for several time windows as the wave depicted in Figure 1a is detected by MESSENGER. The vertical axis represents the wave amplitude in the azimuthal direction, while the horizontal axis represents the poloidal amplitude over each time window. The color of each ellipse represents the handedness of polarization; red corresponds to right‐handed (RH) polarization and green is left handed (LH). The handedness is defined using the dot product of the wave vector, **k**, with the ambient magnetic field vector, **B**, where **k**·**B** > 0 for a right‐hand polarized wave and **k**·**B** < 0 for a left‐hand polarized wave [*Means*, 1972]. The polarization is closest to circular near the magnetopause and becomes linear at around 10:30 during a flip in handedness from LH to RH. After this flip in handedness, the wave briefly becomes more elliptical, until shortly after 10:32, where the wave becomes almost completely linear in polarization. At this time, the polarization handedness reverses again back to LH polarization.

Figure 1d shows the *L* shell and magnetic local time (MLT) of MESSENGER's magnetic equatorial footprint in orange and blue, respectively. It can be deduced from this figure that the wave is observed in the late morning sector around 10:30 MLT, where the magnetic equatorial footprint of MESSENGER traverses planetward. This figure and its remaining panel, Figure 1e, shall be discussed in further detail in section 4.

### 2.3 Magnetic Field Model and Mapping

A number of models of Mercury's magnetosphere have been created using various methods including the modification of Earth‐like models to fit the Hermean magnetosphere [*Luhmann et al.*, 1998; *Sarantos et al.*, 2001; *Korth et al.*, 2004] or based on a simplistic magnetopause shape [*Grosser et al.*, 2004]. More recently, another model was created by *Alexeev et al.* [2008, 2010] that incorporated a paraboloid‐shaped magnetopause, which had previously been successfully developed for the magnetospheres of Earth, Jupiter, and Saturn. Unfortunately, the paraboloid shape of the magnetopause does not agree with the observed magnetopause shape [*Winslow et al.*, 2013]. Also, the paraboloid model contains unrealistic magnetic islands [see *Korth et al.*, 2014] which makes tracing field lines into certain parts of the magnetotail impossible. The most recent magnetic field model is the KT14 [*Korth et al.*, 2015] model, which is the model used in this study. The KT14 model was built using the same modular approach to models made for Earth [see *Tsyganenko*, 2013], where each module contains a magnetic field source (e.g., a current system or the intrinsic field of the planet) which is contained within the magnetopause boundary using a derived magnetopause shielding field. The individual modules and their associated magnetopause fields are then summed together to create the total model field.

**Figure 3:**
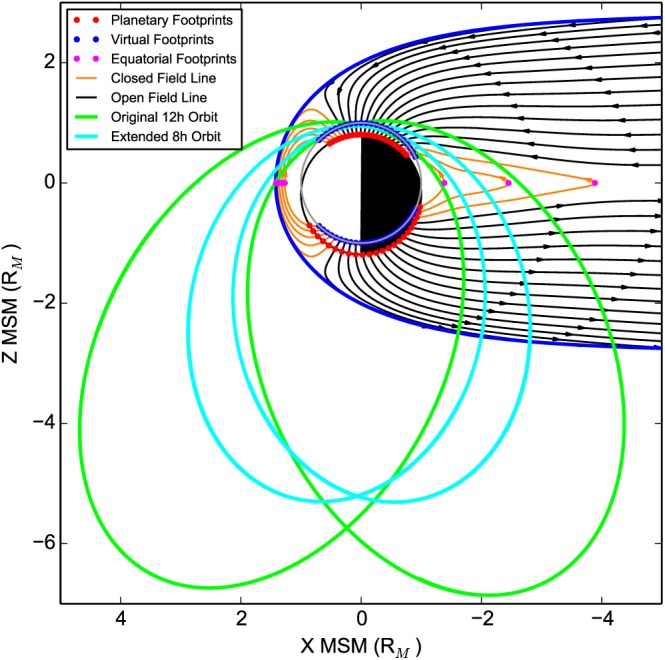
Example magnetic field traces in the *X*‐*Z* MSM plane, where orange lines are “closed” field lines which connect to both hemispheres of the planet and black lines are “open,” with only one planetary footprint, the other being connected to the solar wind. Pink dots represent the magnetic equatorial footprints of the closed field lines, red dots are the footprints on the surface of Mercury, and blue dots are the footprints on the Mercury‐sized virtual sphere (gray line) centered upon the magnetic dipole. MESSENGER's orbital paths for its original 12 h orbit and eventual 8 h orbit are shown in green and cyan, respectively.

For each spectrum found using the technique described above, we used the KT14 field model to map the field lines at MESSENGER's position to a location in the magnetic equatorial plane and to a position on the surface of Mercury. Figure 3 shows some example field line traces performed using the magnetic field model, where black and orange lines are the traces for the open (connected to the IMF) and closed (both ends connected to Mercury) field lines, respectively. The red dots show the locations of the field line footprints on Mercury's surface, and the pink dots are the footprints on the magnetic equatorial plane. Due to the offset of Mercury's dipole by ∼0.196 *R*_*M*_ into the northern hemisphere [*Anderson et al.*, 2011a, 2012; *Johnson et al.*, 2012], we also traced the field lines to a virtual surface, the same size as Mercury, centered upon the planetary dipole—similar to the method used by *Korth et al.* [2014], where each footprint has an invariant latitude and local time. This surface is depicted in Figure 3 by a gray circle centered upon the magnetic dipole; the field line footprints on this surface are marked by blue dots. The use of invariant latitude allows us to directly compare wave activity traced to both the northern and southern hemispheres.

## 3 Results

**Figure 4:**
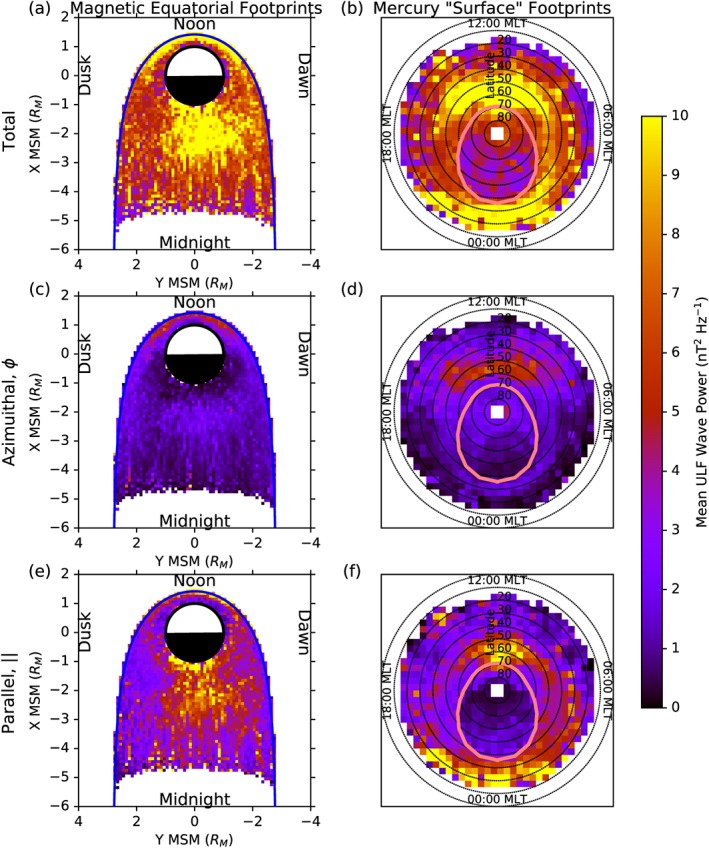
Mean ULF wave power (a, c, and e) traced to the magnetic equatorial plane and (b, d, and f) traced to invariant latitude‐local time coordinates. Figures 4a and 4b show the mean total power, the sum of the azimuthal, parallel, and poloidal powers. Figures 4c and 4d show the mean wave power for the azimuthal component, while Figures 4e and 4f show the mean wave power for the parallel component. Each panel is oriented such that noon is at the top and dawn is to the right. The concentric dotted circles present in Figures 4b, 4d, and 4f represent lines of latitude, each separated by 10°, where 90° is at the center of the axes. The pink oval represents the polar cap boundary as determined using the KT14 magnetic field model.

The distribution of detected wave power is presented in Figure 4, where Figures 4a, 4c, and 4e show the mean wave power traced to the magnetic equatorial plane and Figures 4b, 4d, and 4f show the mean wave power traced to invariant latitude‐local time coordinates on the virtual surface shown in Figure 3. In the panels representing the invariant latitude surface, concentric dotted circles represent every 10° of invariant latitude, where the outermost circle is the equator, and the center of the figure is the pole. The pink oval present in the invariant latitude plots represents the boundary between open and closed field lines as determined using the KT14 magnetic field model. All six panels are oriented such that noon is at the top and dawn is to the right. The top pair of panels (Figures 4a and 4b) shows the mean wave power for the sum of the poloidal, azimuthal, and parallel components, Figures 4c and 4d show the mean azimuthal wave power, and Figures 4e and 4f show the mean parallel wave power. Higher wave powers appear as yellow and orange, while lower wave powers appear as purple and black.

Figures 4a and 4b show that significant wave power maps to all locations within ∼5 *R*_*M*_ of Mercury in the magnetic equatorial plane and to all magnetic latitudes above ∼20°. There is a large concentration of wave power along the dayside magnetopause, which maps to locations between ∼40 and 70° magnetic latitude on the dayside surface. Another large concentration in wave power exists in the nightside of the magnetosphere, slightly dawnward of midnight. This nightside peak in wave power maps to a relatively narrow band of latitudes between ∼15 and 35°. It can be seen in Figure 4b that the majority of the wave power maps to the surface to form an oval, the center of which exhibits a lack of wave power and is displaced toward the nightside of Mercury.

Azimuthally oscillating waves could represent standing Alfvén waves similar to the toroidal waves observed at Earth. Figures 4c and 4d show that the majority of the azimuthal wave power is found close to the dayside magnetopause, forming part of the dayside peak in total wave power seen in Figures 4a and 4b. This region of enhanced azimuthal wave power maps down to magnetic midlatitudes on the surface of Mercury but is much less powerful than the dayside peak in wave power shown in Figure 4b. This suggests that much of the azimuthal wave activity is accompanied by a significant compressional (parallel) component.

The compressional (parallel) component shown in Figures 4e and 4f makes up the largest contribution to the total wave power in Figures 4a and 4b. The nightside peak in particular is predominantly compressional, though a small peak in compressional power is present along the inside of the magnetopause. Figure 4f shows that this component of the wave power is enough to reveal the location of the polar cap boundary discussed above.

While looking at the average wave powers for each component is useful, it does not provide a full picture of what types of waves may exist in a given location. The waves present near the dayside magnetopause which have a large azimuthal component to their wave power may not be purely or even predominantly azimuthal; they may be dominated by a more significant compressional component. In order to compare the three components with each other, three ratios have been defined for each spectral peak detected. These three ratios are defined by

(1) $$\begin{array}{cc} R_{\mathrm{\phi c}} & =\frac{P_{\phi }}{P_{P}+P_{∥}}=\frac{\text{Azimuthal}}{\text{Non‐Azimuthal}},\\ R_{∥⊥} & =\frac{P_{∥}}{P_{P}+P_{\phi }}=\frac{\text{Parallel}}{\text{Transverse}},\\ R_{\mathrm{\phi P}} & =\frac{P_{\phi }}{P_{P}}=\frac{\text{Azimuthal}}{\text{Poloidal}}, \end{array}$$

where *P*_*P*_, *P*_*ϕ*_, and *P*_∥_ are the poloidal, azimuthal, and parallel wave powers.

**Figure 5:**
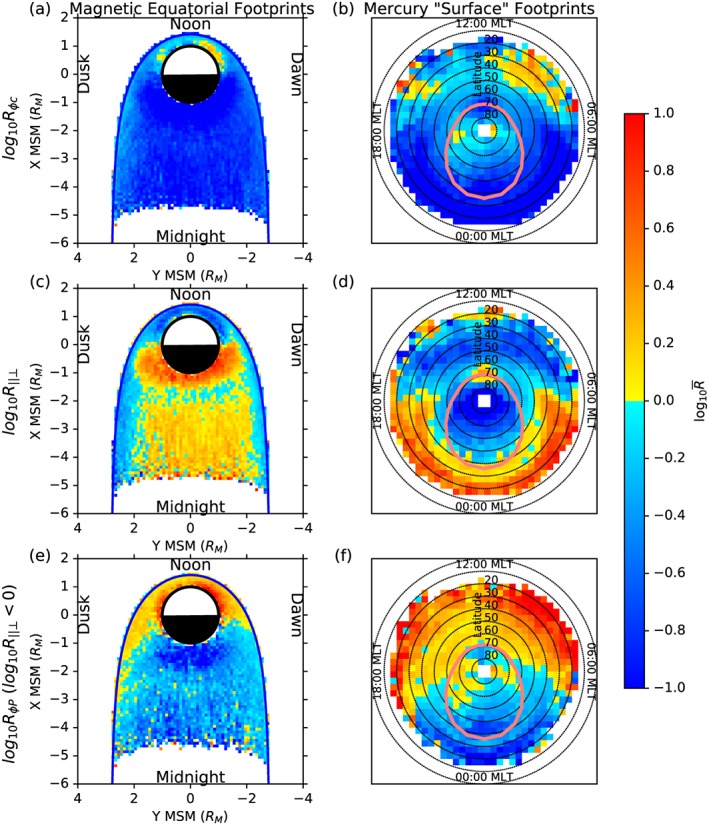
(a, c, and e) Magnetic equatorial footprints and (b, d, and f) invariant latitude footprints, as in Figure 4. Here each spatial bin is the $\underset{10}{\log}$ of the mean of a ratio, where Figures 5a and 5b show the ratio of azimuthal (yellow‐red) to nonazimuthal wave power (blue) and Figures 5c and 5d show the ratio of parallel (yellow‐red) and transverse (blue) wave power. Figures 5e and 5f show the ratio of the azimuthal (yellow‐red) and poloidal (blue) components of the wave power for just the transverse‐dominant waves; the parallel dominant waves were discarded for this comparison.

The mean of the logarithm of each of these three ratios is presented in Figure 5, where Figures 5a, 5c, and 5e show the data traced to the magnetic equatorial plane and Figures 5b, 5d, and 5f show the data mapped to invariant latitude and local time in the same format as in Figure 4. Figures 5a and 5b show the spatial distribution of $\underset{10}{\log}R_{\mathrm{\phi c}}$, where values above zero in yellow or red represent areas where most waves are dominated by their azimuthal component, and negative values in blue are where the nonazimuthal components dominate. Waves with a predominantly azimuthal polarization are most common on the dayside of the planet, particularly in the late morning sector, while the rest of the magnetosphere seems to be dominated by the other two components. Figure 5a shows that the small azimuthally dominant areas exist very close to the planet, even though the azimuthal wave power is most abundant near the magnetopause at similar local times in Figures 4c and 4d. This may indicate that waves with mixed polarizations, but with a significant azimuthal component, near to the magnetopause could be driving more azimuthally oscillating wave activity closer to the planet, mapping to latitudes slightly equatorward of the polar cap boundary.

Figures 5c and 5d show the average of $\underset{10}{\log}R_{∥⊥}$, which compares the parallel compressional power (>0, red and yellow) to the transverse wave power (<0, blue and cyan). Transverse wave power is the combination of the poloidal and azimuthal components of wave power and is dominant near to the magnetopause, particularly on the dayside of the magnetosphere. Compressional waves are most common in the nightside inner magnetosphere and throughout the magnetotail. It is likely that the transverse dominance near the magnetopause is related to the K‐H interaction with the magnetosheath or another antisunward propagating mechanism.

In Figures 5e and 5f, the average of $\underset{10}{\log}R_{\mathrm{\phi P}}$ ratio is presented for the transverse‐dominated population of waves ( $\underset{10}{\log}R_{∥⊥}<0$, no compressionally dominant waves). This is a direct comparison between the two transverse components, where positive values in red and yellow represent areas of azimuthally dominant wave activity and negative values in blue and cyan represent areas of poloidal wave dominance. Of the transverse wave population, predominantly azimuthal oscillations are most common throughout the entire dayside magnetosphere and much of the dusk flank, where poloidal waves are most common elsewhere, particularly close to the nightside of Mercury. The dawn‐dusk asymmetry present in these figures could be related to the dawn‐dusk asymmetry in the K‐H magnetopause waves observed by MESSENGER [*Sundberg et al.*, 2012b].

**Figure 6:**
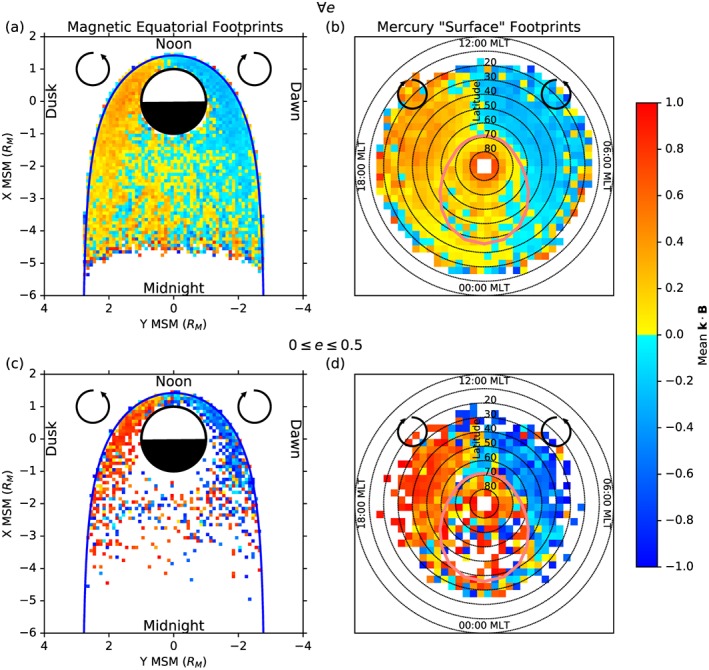
(a and b) The mean **k**·**B** for all of the transverse waves mapped to the magnetic equatorial plane and invariant latitude, respectively. (c and d) The same as Figures 6a and 6b except that only the most circular waves with eccentricities in the range 0≥*e*≥0.5 were used. Positive **k**·**B** (yellow‐red) represents right‐handed wave polarization, and negative **k**·**B** (blue) represents left‐handed polarization.

The transverse population of waves can be studied further in terms of their eccentricity and polarization handedness. Most of the waves detected in this study exhibited near‐linear polarization, though a small percentage had eccentricities of *e* < 0.5. The handedness of the wave polarization is calculated from the dot product of the wave propagation vector with the ambient magnetic field vector, **k**·**B**, as discussed in section 2.2. Figure 6 shows the average values of **k**·**B** in the equatorial plane (Figure 6a) and invariant latitude‐local time (Figure 6b) for all eccentricities, while Figures 6c and 6d show the same thing for waves with *e* < 0.5, the most circularly polarized waves. It is clear from all four panels that there is a flip in the average wave handedness near to noon, regardless of how linear the polarization. Generally, right‐handed (RH) polarized waves, in red and yellow **k**·**B** > 0, occur on the duskside of the magnetosphere, and left‐handed (LH) waves, in blue and yellow **k**·**B** < 0, are observed on the dawnside. This switch in polarization is most notable with the most circularly polarized events, which have the clearest polarization signatures. It is interesting to note that the most circular waves occur almost exclusively along the magnetopause and that the direction in which they are polarized is suggestive that the magnetosheath flow past the magnetopause has imparted this polarization upon them.

## 4 Discussion

The distribution of wave power throughout the magnetosphere, as presented by Figure 4, shows that much of the power is concentrated in two regions: the first is just within the dayside magnetopause and the second in the near magnetotail, ever so slightly skewed toward dawn. The concentration of wave power, particularly azimuthal wave power, close to the dayside magnetopause indicates that solar wind interaction with the magnetopause could be a major source of ULF wave activity at Mercury. Compressional wave activity, while common throughout the entire magnetosphere, is mostly responsible for the region of high wave power in the magnetotail.

When traced to invariant latitude, the compressional wave activity appears to be concentrated to a ring of high wave power, forming an oval around lower average wave powers. This oval is most clear in the total power, where there is a significant lack of wave power present within the oval. The boundary between high and low wave power is almost identical in location to the polar cap boundary predicted by the KT14 model. This suggests that the wave power outlines the average polar cap location such that equatorward of the boundary, standing waves exist on closed field lines, bouncing between hemispheres, and poleward of the boundary, standing waves cannot form as they are on open field lines.

Figures 5e and 5f show that when the compressional waves are excluded, the entire dayside magnetosphere and flanks are dominated by azimuthally oscillating ULF waves. The level of this dominance of azimuthally oscillating wave activity is at its highest very close to the planet and provides evidence to suggest that the interaction with the solar wind could be capable of driving toroidal field line resonances similar to those observed in the terrestrial system.

The polarization of these transverse waves, shown in Figure 6, exhibits a clear reversal around the noon‐midnight meridian. The handedness of the waves on each side of the magnetosphere suggests that they inherited their polarization state from antisunward propagating features of the solar wind such as K‐H magnetopause waves. The wave activity that this interaction is expected to induce is well known in the case of the Earth's magnetosphere, where resonant, antisunward traveling toroidal mode waves are induced by the presence of magnetopause surface waves on the flanks of the magnetosphere. In Mercury's multicomponent plasma environment, it may still be possible for resonant mode coupling to occur in this way with the shear Alfvén mode as long as the frequency is significantly lower than that of the lowest ion gyrofrequency; otherwise, localized coupling may be present at the crossover frequency.

The frequency of a shear Alfvén mode resonance depends on the length of the field line, *L*, and the Alfvén speed, *v*_*A*_, where

(2) $$v_{A}=\frac{B}{\sqrt{\mu_{0}\rho }},$$

*B* is the magnetic field strength and *ρ* is the plasma mass density. The wave period can then be expressed as a time‐of‐flight calculation,

(3) $$T=2\overset{L}{\underset{0}{\int }}\frac{1}{v_{A}}\text{d}l$$

where d*l* is an infinitesimal element of the total field line length, *L* [*Denton and Gallagher*, 2000; *Chi and Russell*, 2005; *Takahashi et al.*, 2014]. This can be approximated by a summation over a finite number of steps along the field line,

(4) $$T=2\overset{n}{\underset{i}{\sum }}\frac{l_{i}\sqrt{\mu_{0}\rho_{i}}}{B_{i}}.$$

The crossover frequency, *f*_CR_, is dependant upon the local magnetic field strength, |**B**|, and the relative concentrations of the constituent ion species. For a three‐component plasma, where the frequency is far below the electron gyrofrequency, the crossover frequency can be expressed by

(5) $$f_{\text{CR}}={\left(p_{1}\frac{Z_{2}^{2}}{m_{a2}}+p_{2}\frac{Z_{1}^{2}}{m_{a1}}\right)}^{\frac{1}{2}}\frac{e|B|}{2\mathrm{\pi u}},$$

where *p*_*i*_, *Z*_*i*_, and *m*_*ai*_ are the relative concentration fraction, charge state, and the atomic mass of the ion species *i*, *e* is the elementary charge, and *u* is the unified atomic mass unit. The concentration fraction of a given ion species, *p*_*i*_, is calculated using $p_{i}=\frac{n_{i}}{n_{e}}$, where *n*_*i*_ and *n*_*e*_ are the number densities of species *i* and electrons, respectively, and *p*_1_+*p*_2_=1. The crossover frequency must always be present somewhere between the gyrofrequencies of ion species 1 and 2 and exists closest to the species with the smallest *p* value.

**Figure 7:**
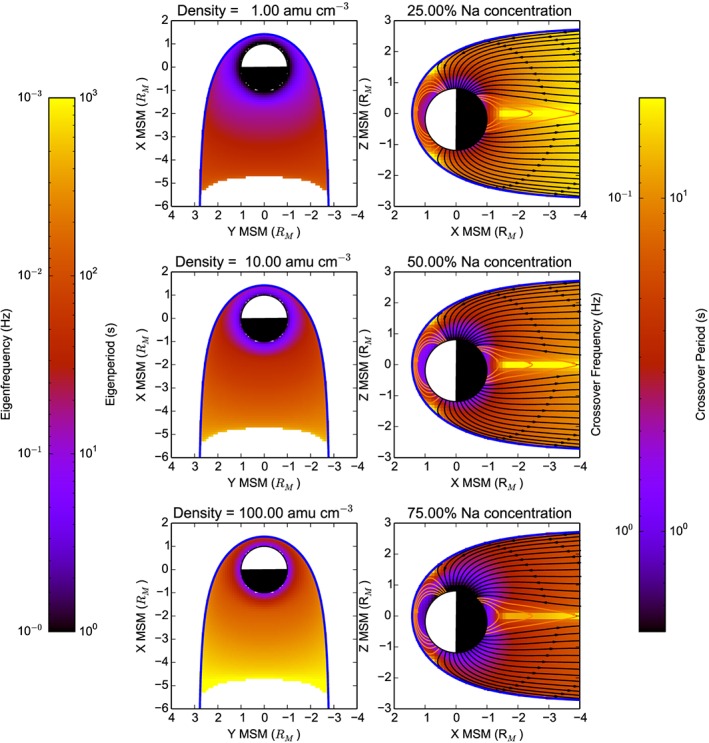
Magnetospheric maps of (left column) modeled toroidal eigenfrequencies, *f*_FLR_, in the *X*‐*Y* MSM plane and (right column) crossover frequencies in the *X*‐*Z* MSM plane. From top to bottom, the left panels show the eigenfrequencies mapped to the equatorial plane assuming uniform plasma densities of 1, 10, and 100 amu cm^−3^. The top, middle, and bottom panels on the right show the crossover frequency, *f*_CR_, based on uniform Na^+^ to H^+^ concentration ratios of 25, 50, and 75%, respectively. Eigenfrequencies (eigenperiods) range from 1 mHz to 1 Hz (1 to 1000 s), and crossover frequencies range from 50 mHz to 2 Hz (0.5–20 s), where lowest wave frequencies are expressed in black and purple and higher frequencies are represented by yellow and red.

Using equations (4) and (5) alongside the KT14 magnetic field model to provide the field strength at any given location within the magnetosphere, and to estimate field line lengths, it is possible to model the frequencies/wave periods expected to be present at Mercury. Figure 7 shows the resonant frequencies expected for shear Alfvén waves in the left column assuming a uniform plasma density of 1, 10, and 100 amu cm^−3^ (top to bottom) in the *X*‐*Y* MSM plane and the crossover frequencies for 25, 50, and 75% (top to bottom) sodium concentrations in the *X*‐*Z* MSM plane on the right column. For all modeled plasma mass densities, the FLR eigenfrequency is highest on the shortest field lines, closest to Mercury and lowest on the longest field lines stretching out into the magnetotail. The FLR eigenfrequency is highest for lower plasma mass densities, reaching ∼1 Hz close to the surface of Mercury in the lowest modeled density of 1 amu cm^−3^ but may also be as low as ∼1 mHz for much higher modeled densities, on longer field lines. The predicted crossover frequencies are generally higher than the FLR eigenfrequencies throughout the magnetosphere. The highest crossover frequencies would be expected closest to Mercury, where field strength is the strongest, and lowest in regions of low field strength. Depending on relative sodium concentration, crossover frequencies close to the planet would be expected to reach >1 Hz, which is similar to the local FLR eigenfrequency for very low plasma mass densities.

It is possible that the solar wind‐related wave activity evident in Figures 4 and 6 could couple with toroidal FLRs or the local crossover frequency. Figure 7 provides an idea of how the frequencies of both types of resonance may vary depending on the location within the magnetosphere. In the case where toroidal FLRs were common, azimuthally oscillating ULF waves should be excited at higher frequencies on shorter field lines, which map to lower *L* shells. We may also expect that while the wave power reduces with distance from the magnetopause, there may be a peak in average wave power at a location deeper in the magnetosphere where resonance may be common. For Earth‐like FLRs we would expect to observe a flip in polarization handedness at smaller radii in Figure 6, around the location of the resonant field line. The lack of evidence of such a reversal could be explained by either a very variable resonance location or relatively poorly formed resonances where the polarization reversals are not completely obvious. Such resonances have been modeled at Earth for some combinations of wave scale length, damping, and Alfvén speed gradients [e.g., *Hughes and Southwood*, 1976]. Alternatively, if the wave activity is coupling with the local crossover frequency, we could expect a peak in transverse wave power that is ordered with the local ambient magnetic field magnitude and that lies between the hydrogen and sodium gyrofrequencies.

**Figure 8:**
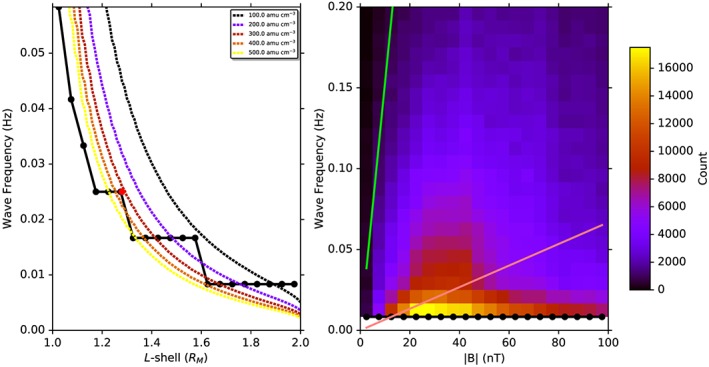
(left) Modal observed frequency for 20 *L* shell bins between *L* shells of 1.0 and 2.0 *R*_*M*_. Dashed lines represent the frequency profiles, *f*_FLR_, of resonant field lines that have equatorial footprints at 06:00 or 18:00 MLT for five densities from 100 to 500 amu cm^−3^. The red dot represents where the wave presented in Figure 1 exists. (right) The modal observed frequency for 20 magnetic field magnitude bins between 0 and 100 nT. The number of spectra present in each bin is presented in color. The pink and green lines represent the gyrofrequencies of sodium and hydrogen ions, respectively.

Figure 8 (left) shows the modal azimuthally dominant wave frequency as a function of *L* shell taken from all magnetic local times. For comparison, the expected eigenfrequencies for densities of 100–500 amu cm^−3^ at 06:00 or 18:00 MLT are displayed as dashed lines. The red dot present in the figure will be discussed later. The step‐like nature of modal frequencies represented by the solid line in this figure is likely to be an artifact created by the finite size of the frequency bins in the output of the FFT. While the modal frequency does not follow a single density line, it does increase at lower *L* shells as would be expected if these waves were FLRs. The dashed curves also suggest that there may be an increase in plasma density closer to Mercury's surface.

Because the crossover frequency increases with magnetic field strength, if it were to exist within the frequency range of this study, it would occur at relatively low |**B**|. Figure 8 (right) shows the modal frequency (black line with black dots) against magnetic field strength, with the number of spectral peaks at each frequency and magnetic field strength bin in color in the background. The pink and green lines represent the sodium and proton gyrofrequencies, respectively. The modal frequency does not appear to change with magnetic field strength and typically lies far below the lowest ion gyrofrequency. There is some evidence that at low magnetic field strengths (<50 nT), there is some ordering with |**B**|. This could be evidence of ion‐cyclotron waves at the local sodium gyrofrequency, and a small number of IIH resonances at the crossover frequency, between the two gyrofrequencies. It appears that this is not the preferred form of resonance at low frequencies, and close to the planet, where the waves selected for analysis here have frequencies far below that of the local sodium gyrofrequency.

**Figure 9:**
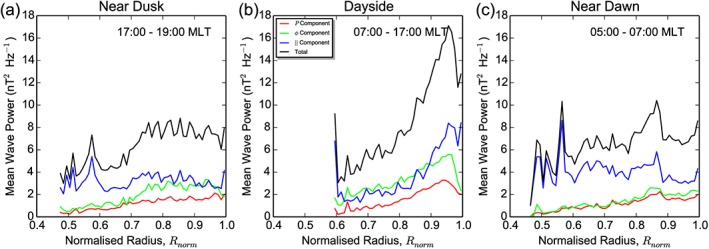
Total (black), poloidal (red), azimuthal (green), and parallel (blue) wave power against normalized radius for (a) dusk, (b) dayside, and (c) dawn. Normalized radius is defined by *L*/*R*_MP_, where *L* is the *L* shell of MESSENGER's equatorial footprint and *R*_MP_ is the radius of the magnetopause at a given local time.

Figure 9 shows how the average wave power varies with distance from the magnetopause near dusk (a), through most of the dayside (b), and near dawn (c). The average power for the poloidal, azimuthal, and parallel components and the sum of all three components are presented in red, green, blue, and black, respectively. The power is plotted against normalized radius, which is the radial distance of the equatorial footprint of the wave, divided by the radial distance of the magnetopause at that local time, so *R*_norm_=1.0 represents the magnetopause and *R*_norm_=0 is the center of the planet. If a resonance condition was a common occurrence in a given region, it might be expected that a peak in wave power should appear in that location. The azimuthal wave power does not appear to show any significant peaks in any of the panels of Figure 9, possibly suggesting that toroidal field line resonances may be relatively uncommon. Unlike the power profiles for the azimuthal and poloidal wave powers, the compressional wave power has several peaks deep into the magnetosphere where 0.5 < *R*_nom_<0.6, near both dawn and dusk. This suggests that a highly compressional resonance may be present at Mercury, possibly driven by activity on the flanks of the magnetosphere.

A uniform magnetospheric plasma density is obviously very unlikely, but the three mass densities modeled above, in Figure 7, could be fairly representative of various different regions of the magnetosphere. During Mariner 10's first and third flybys of Mercury, it measured the density of the cool plasma sheet to be 3–7 protons cm^−3^ [*Ogilvie et al.*, 1977]. *Raines et al.* [2011] estimated proton densities in the magnetotail using Fast Imaging Plasma Spectrometer (FIPS) [*Zurbuchen et al.*, 1998; *Andrews et al.*, 2007] of 1–20 cm^−3^ during MESSENGER's M1 and M2 flybys, where sodium ion densities were calculated to be approximately 1 cm^−3^ in order to make up for missing magnetic pressure. Heavy ions observed using FIPS had very low average densities of 3.9 × 10^−2^ cm^−3^ for He^2+^, 3.4×10^−4^ cm^−3^ for He^+^, 8.0×10^−4^ cm^−3^ for O^+^, and 5.1 × 10^−3^ cm^−3^ for Na^+^ [*Raines et al.*, 2013], though sodium densities were found to be higher in the cusps (up to 2 cm^−3^) [*Raines et al.*, 2014] and the premidnight sector.

Plasma mass densities in Mercury's magnetosphere have also been modeled in a number of simulations. *Benna et al.* [2010] used a multifluid model to study the Hermean magnetosphere during the first MESSENGER flyby. This model predicted the existence of a drift belt at <1.6 *R*_*M*_ from the center of Mercury with proton densities of 8–10 cm^−3^. In the morning sectors, proton densities reached >10 cm^−3^, while the cusps hosted proton densities from 10 to 100 cm^−3^. Simulations have also predicted the density of sodium ions within the magnetosphere [*Leblanc et al.*, 2003; *Delcourt et al.*, 2003; *Yagi et al.*, 2010], where densities typically peak in the dayside magnetosphere at 10–100 sodium ions cm^−3^ but are much lower in the nightside. This number of sodium ions would provide the majority of the mass density on the dayside of the magnetosphere. Overall, modeled plasma densities are expected to be in the range of ∼200–2000 amu cm^−3^ in the dayside magnetosphere and less than 200 amu cm^−3^ in the nightside magnetosphere.

In the case where the frequency of the wave is known, it is possible to work backward to estimate the plasma density given an assumption of how the plasma mass density varies along the field line. Figure 1e shows the calculation in equation (4) reversed in order to estimate the plasma density. For this calculation, field line length and field strength were obtained using the KT14 model field traces from MESSENGER's position to the surface of Mercury, and plasma mass density was assumed to be constant along the field line. The calculation has been performed for all times during the event regardless of whether there was resonance at the time. At the approximate time of resonance (∼10:32:17 UT), the calculation yields a plasma mass density of ∼240 amu cm^−3^, which is consistent with the models mentioned above. This event is also represented in Figure 8 (left) by a red dot and appears to be very characteristic of the other azimuthally oscillating waves at a similar *L* shell.

## 5 Conclusions

In this study of ULF wave activity, power, polarization, and frequency have been characterized on a global scale in the Hermean magnetosphere. Observations show that wave power is common throughout the magnetosphere and that compressional waves provide more of this wave power than the azimuthal or poloidal waves. Azimuthal wave power is most common within the dayside magnetopause, providing evidence that interactions with the solar wind such as the Kelvin‐Helmholtz instability may be driving ULF wave activity within the magnetosphere, possibly through field line resonance. Compressional wave power was present everywhere but peaked near midnight, close to the planetary surface. The wave power also traced out the likely location of a polar cap boundary in agreement with the KT14 magnetic field model, where very little wave activity occurs on the open field lines poleward of the boundary, and large amounts of wave activity are present on the closed field lines equatorward of this boundary.

Further evidence that solar wind interactions could be driving wave activity is found when studying the polarization direction of transverse ULF waves. The average polarization direction is left handed on the dawnside and right handed on the duskside of the magnetosphere, as if their polarization is inherited from the antisunward flow of features within the magnetosheath. This is most distinct with the more circular wave population, which exists closest to the magnetopause.

While there is little evidence to suggest that this interaction with the solar wind is driving wave activity at the crossover frequency, there is some evidence that there may be coupling with field line resonances. The azimuthally dominant wave activity tends to decrease in frequency on field lines with larger *L* shells—where the field line length would be longer. The lack of evidence for resonances at the crossover frequency is because the crossover frequency would only be visible in the frequency band studied here at large distances from Mercury, where field strength is lower. Resonances at the crossover frequency may be more common at higher frequencies, closer to the planet, which could be the subject of future research.

One example ULF wave observed within the dayside magnetopause exhibits polarization changes somewhat consistent with field line resonance theory at Earth. Using the simple assumption of a constant plasma density, a time‐of‐flight calculation is reversed to estimate a plasma mass density of ∼240 amu cm^−3^. This density is far higher than the average densities measured using FIPS [*Raines et al.*, 2011, 2013, 2014] but is very consistent with modeled sodium ion densities [*Leblanc et al.*, 2003; *Delcourt et al.*, 2003; *Yagi et al.*, 2010]. More events similar to the example event presented here may represent FLR activity and could be a useful tool to provide further density estimates within the Hermean magnetosphere.
